# Short-Term Adaptation of Dairy Cattle Production Parameters to Individualized Changes in Dietary Top Dress

**DOI:** 10.3390/ani11123518

**Published:** 2021-12-10

**Authors:** Tanner P. Price, Vinícius C. Souza, Douglas M. Liebe, Mark D. Elett, Ty C. Davis, Claire B. Gleason, Kristy M. Daniels, Robin R. White

**Affiliations:** 1 Department of Animal and Poultry Sciences, Virginia Tech, Blacksburg, VA 24061, USA; tprice24@vt.edu (T.P.P.); liebe2@vt.edu (D.M.L.); dtyc@vt.edu (T.C.D.); colubr1d@vt.edu (C.B.G.); danielsk@vt.edu (K.M.D.); 2 Department of Dairy Science, Virginia Tech, Blacksburg, VA 24061, USA; vinicius2042@hotmail.com (V.C.S.); emark4@vt.edu (M.D.E.)

**Keywords:** short-term, individualized, precision, feeding

## Abstract

**Simple Summary:**

“Short-Term Adaptation of Dairy Cattle Production Parameters to Individualized Changes in Dietary Top Dress” focuses on feeding dairy cattle varying amounts of top dressed corn grain, soybean meal, or grass hay in order to obtain data on individual cow responses to be utilized for future development of individualized dairy precision feeding models. Precision feeding systems aimed at feeding dairy cattle individually to increase feed efficiency could contribute to profitability of dairies. The information provided in this study includes considerations of how we should design such systems.

**Abstract:**

Immediate and short-term changes in diet composition can support individualized, real-time interventions in precision dairy production systems, and might increase feed efficiency (FE) of dairy cattle in the short-term. The objective of this study was to determine immediate and short-term effects of changes in diet composition on production parameters of dairy cattle fed varying amounts of top dressed commodities. A 4 × 4 replicated Latin square design was used to evaluate responses of twenty-four Holstein cows fed either no top dress (Control) or increasing amounts of: corn grain (CG), soybean meal (SBM), or chopped mixed grass hay (GH) top dressed on a total mixed ration (TMR) over four, 9-day periods. Throughout each period, top dressed commodities were incrementally increased, providing 0% to 20% of calculated net energy of lactation (NE_L_) intake. Measured production responses were analyzed for each 9-d period using a mixed-effects model considering two different time ranges. Samples collected from d 3 and 4 and from d 7 and 8 of each period were averaged and used to reflect “immediate” vs. “short-term” responses, respectively. In the immediate response time frame, control fed cows had lower milk yield, milk fat yield, and milk true protein yield than CG and SBM supplemented animals but similar responses to GH supplemented animals. Milk fat and protein percentages were not affected by top dress type in the immediate term. In the short-term response time-frame, GH supplemented animals had lower DMI and milk fat yield than all other groups. Control and GH supplemented cows had lower milk yield than CG and SBM fed cows. In the immediate response time frame, FE of SBM supplemented cows was superior to other groups. In the short-term time frame, FE of GH and SBM groups was improved over the control group. Results suggest that lactating dairy cows show rapid performance responses to small (<20% NE_L_) changes in dietary composition, which may be leveraged within automated precision feeding systems to optimize efficiency of production. Before this potential can be realized, further research is needed to examine integration of such strategies into automatic feeding systems and downstream impacts on individual animal FE and farm profitability.

## 1. Introduction

Precision dairy feeding systems may have the ability to optimize individual cow feed efficiency (FE) and milk yield (MY), while decreasing labor expenses [1]. These precision feeding systems may be automated for efficiency of on-farm labor and be individualized to feed each cow a unique supplement. This approach to individualized feeding has the opportunity to maximize each cow’s FE, MY, and milk components of interest based on the unique genetic merit of individual animals. It is now commonplace for dairy farmers to collect multiple forms of data on individual cows daily [2]. However, these data are rarely compiled and utilized to make real-time improvements in individual cow performance. The various information many dairy farmers already collect could be utilized in advanced algorithms to maximize FE and production. Through these automated, individualized, and data-based feeding approaches, precision feeding technologies may allow a beneficial opportunity to tailor dietary supplements to individual cows more precisely. However, we lack data to quantify this potential benefit and to define appropriate and necessary decision-making algorithms.

Developing accurate dairy precision feeding systems is a slow process due to the lack of available data on individual dairy cows necessary to inform algorithms about what types of feeding strategies should be implemented when using automated feeders for individual cows. Indeed, Wolfert, et al. [3] reported that on-farm data generally remain in the hands of individual companies, which present a challenge to develop algorithms to be used in precision feeding systems. However, if collections of data are generated and integrated and appropriate algorithms are derived from these data, increases in feed efficiencies and MY may provide additional profit for farmers. In the long-term, favorable outcomes such as these might outweigh initial costs and labor expenses associated with automated feeding technologies.

Automated feeding systems may have difficulty handling and dispensing large quantities of feed in the form of a total mixed ration (TMR). Therefore, feeding only a small portion of the diet by an automated system in the form of a supplement is likely the most cost effective and efficient method of automated feeding for dairy operations. This feeding style is effectively the same approach that in-parlor feeding systems and automatic milking systems (AMS) use, but these systems do not generally supplement feeds to cater to the animal’s unique nutrient requirements. Instead, most existing in-parlor feeding systems and AMS feed cows have a partially mixed ration (PMR), as opposed to a TMR, that is balanced to meet the nutritional needs of the average cow in the group of cows. Palatable concentrates are usually fed in existing in-parlor feeding systems and AMS, and forage is typically fed at a separate feed bunk, thus defining a PMR feeding system.

Providing an individual supplement to each cow raises questions such as: what should the supplement be composed of, and when should the composition or quantity offered change? Allen [4] summarized that very specific physical and chemical characteristics of diets can affect DMI in the short-term. Another challenge is that nutrition experiments often utilize adaptation periods, i.e., 14 d on average [5], prior to any data collection. This practice is not useful for studying the impact of diet composition changes on immediate and short-term lactational performance. Therefore, data from these types of experiments cannot be utilized to develop models and algorithms that predict effects of diet composition on lactation performance in the short-term and in real-time. Determining what supplement compositions would benefit a specific cow the most, how much of an individual supplement should be fed on a daily basis, and when to adjust these supplements will require new types of experimentation and data collection to enable development of decision support tools and automated feeding algorithms. Therefore, the objective of this experiment was to determine the immediate and short-term effects of changes in diet composition on FE, MY, and milk components in lactating dairy cows fed increasing amounts of top dressed supplemental feeds. 

## 2. Materials and Methods

### 2.1. Animals, Housing, and Diets

This experiment was conducted from 4 February 2019 to 26 March 2019 (inclusive of the Calan gate adaptation and experimental periods). 

**Figure 1:**
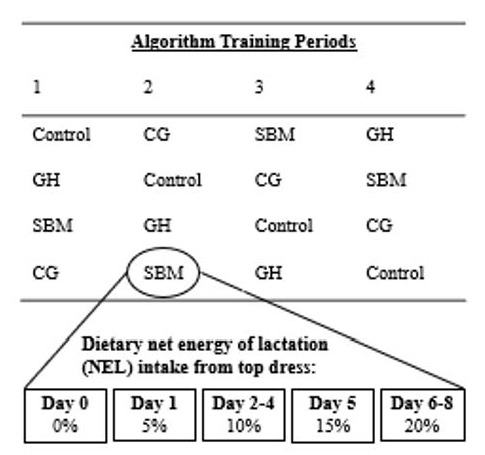
The experimental design consisted of four periods that were nine days in length. During each period, six cows were randomly assigned to one of four treatment options. Experimental treatments included: Control = TMR with no top dress; CG = TMR with corn grain top dress; SBM = TMR with soybean meal top dress; and GH = TMR with mixed grass hay top dress. A replicated 4 × 4 Latin square was implemented and each group of six cows was fed a different experimental treatment diet over the four periods. Cows were fed increasing amounts of dietary net energy of lactation (NE_L_) intake from top dress over each period, as shown in the rectangles above.

Twenty-four Holstein dairy cows (12 primiparous and 12 multiparous; 597 ± 59 kg of BW; and 178 ± 23 DIM at the beginning of the study) were randomly assigned to 1 of 4 treatments in a replicated 4 × 4 Latin square design (Figure 1).

Cows were housed in a 16-stall pen within a free-stall barn and fed once daily (1300 h) using a Calan gate system (American Calan Inc., Northwood, NH, USA). Cows were allowed a 14 d adaptation period before beginning the experiment wherein they were trained to locate their assigned Calan door; this period also allowed ample time for social group reconstruction [6].

A 36-d experimental period followed the adaptation period and consisted of four, 9 d treatment periods. During each period, cows were assigned to one of four top dress treatments: corn grain (CG), soybean meal (SBM), chopped mixed grass hay (GH), or no top dress (Control). Figure 1 details the overall experimental design of this study. A common TMR was fed as the base for all diets and was composed mainly of: corn silage, corn grain, brewers grain, and soybean hulls (Table 1). 

Diets were formulated to be isoenergetic in concentration and to meet or exceed the requirements of the individual cows utilized throughout the experiment [7]. The formulated composition of the common TMR fed to all treatment groups is detailed in Table 1. 

The chemical composition of each top dress and TMR is detailed in Table 2.

The quantity of material top dressed daily varied across each 9-d period and is summarized in Table 3 (net energy basis) and Table 4 (DM basis, kg/d). The term “top dress” used in this study refers to partial (isoenergetic) substitution of CG, SBM, or GH in place of TMR allocated daily.

On days 0, 1, and 2 of each period, the top dress was gradually increased in quantity to provide 0%, 5%, and 10% of dietary net energy of lactation (NE_L_) intake, respectively. On days 3 and 4, the top dress quantity was targeted to provide 10% of dietary NE_L_ (Table 3). On days 5 and 6, the top dress quantity was gradually increased again to provide 15% and 20%, respectively, of the calculated dietary NE_L_. Days 7 and 8 retained the top dress inclusion rate at the target of 20% of dietary NE_L_ (Table 3). As each period progressed and increasing amounts of top dress were provided, correspondingly less TMR was offered (Table 4). Cows on the Control treatment were fed TMR ad libitum. Over the 9 d treatment periods, all cows were fed ad libitum and the quantity of feed offered daily to each cow was calculated based on their individual nutrient requirements, estimated from their performance over the previous day. This value was scaled up or down depending on the individual refusals of each cow from the previous day to target approximately 2 to 5 kg (as-fed basis) in daily refusals for each cow in order to ensure ad libitum feeding without excess waste. All cows had ad libitum access to water and salt (NaCl) throughout the experiment. Animal health and disposition were monitored daily throughout the study.

### 2.2. Feeding Procedure

Cows were fed once daily, coinciding with their afternoon milking. Daily refusal sampling began at 1:00 p.m. A Calan data ranger (American Calan Inc., Northwood, NH, USA) was used to vacuum and remove the refusals from each feed bunk. The Calan data ranger weighed the refusal amounts from each bunk and individual refusal weights for each cow were recorded daily. All cows were taken to the parlor for their afternoon milking while daily refusal sampling and feeding occurred, and returned to their pen at approximately 2:00 p.m. daily, and had access to fresh water. The target amount of TMR for each cow was then dispensed into each feed bunk using the Calan data ranger. The TMR was dispensed within approximately 2.5 kg (as-fed basis) in either direction of the target TMR amount and the exact amount of TMR added to each bunk was recorded. Supplemental top dresses for each cow were weighed into buckets by-hand on a digital platform scale (Defender 5000 XtremeW, model T51XW; Ohaus Corp., Parsippany, NJ, USA). The supplemental top dresses were then thoroughly mixed by hand in order to incorporate all of the top dress into the top third of TMR in each feed bunk. The daily feeding procedure was finished at approximately 1530 h, when the cows had access to the Calan gates.

### 2.3. Milking Procedure

All cows were milked twice daily, approximately every 12 h, in a double 12 De Laval parallel parlor (Dairymen Specialties, Inc., Harrisonburg, VA, USA) equipped with an inline AfiMilk MPC Milk Meter (Afimilk Ltd; Kibbutz Afikim, Israel) for monitoring individual cow MY, and an AfiLab Milk Analyzer (Afimilk Ltd; Kibbutz Afikim, Israel) for inline individual cow milk composition analysis (fat, true protein, lactose). Milk yield and composition data were collected on each cow twice daily as the cow’s radio-frequency identification (RFID) tag was read by the system. This information was stored in AfiFarm dairy farm management software (Afimilk Ltd; Kibbutz Afikim, Israel). Milk lactose data did not correspond to biologically sensible values, suggesting calibration errors in the AfiLab system. As a result, we have elected not to include milk lactose results. Energy-corrected milk (ECM) was calculated according to the following formula: ECM (kg/d) = kg of milk × [(38.3 × % fat × 10 +24.2 × % true protein × 10 + 16.54 × % lactose × 10 + 20.7)/3140] [8].

In addition to in-line milk sampling, monthly milk samples were collected and analyzed as part of routine DHI testing (Lancaster Dairy Herd Improvement Association; Manheim, PA, USA). Milk fat and protein data are summarized in Table S1.

### 2.4. Feed Sample Collection and Analysis

Samples of fresh TMR, fresh GH, and individual cow feed refusals (approximately 500 g, as-fed basis) were collected daily and frozen at −20 °C. After completion of each period, fresh TMR, fresh GH, and individual cow feed refusals were pooled per each 9-d period. Samples of CG and SBM (Big Spring Mill Inc., Elliston, VA; USA) were collected once at the beginning of the experiment because only one load of each feedstuff was utilized in the experiment. 

All feed samples were analyzed for dry matter (DM), crude protein (CP), neutral detergent fiber (NDF), acid detergent fiber (ADF), ash, ether extract (EE), starch, and acid detergent lignin. Prior to analyses of chemical composition, all samples were dried to constant weight in a 55 °C forced-air oven (Thermo Scientific Heratherm Advanced Protocol Ovens Model 51028115; Fisher Scientific) for 48 h and ground with a Model 4 Wiley mill (A. H. Thomas Scientific, Swedesboro, NJ, USA) to pass through a 1-mm screen. Crude protein was calculated as N x 6.25 after quantification of total N by combustion analysis (Vario El Cube CN analyzer, Elementar Americas Inc., Ronkonkoma, NY, USA). The NDF and ADF were analyzed following the methodology described by Van Soest, et al. [9] and adapted for the ANKOM 200 fiber analyzer (ANKOM Technology, Macedon, NY, USA). We used α-amylase and sodium sulfite for NDF analysis. Ash concentrations were determined in a muffle furnace at 500 °C for 8 h. Ether extract was analyzed by using the Ankom XT10 with petroleum ether according to the manufacturer’s recommendations. Starch concentrations were determined using the acetate buffer method with α-amylase from *Bacillus licheniformis* (FAA, Ankom Technology, Macedon, NY, USA) and amyloglucosidase from *Aspergillus niger* (E-AMGDF, Megazyme International, Wicklow, Ireland). Acid detergent lignin was analyzed in ADF residues using 72% sulfuric acid on a rocking platform (Flask Dancer, Boekel Scientific, Feasterville-Trevose, PA). The chemical composition of individual feedstuffs, including TMR, CG, SBM, and GH, are shown in Table 2.

### 2.5. Statistical Analysis

Outcomes of interest included: DMI, MY, milk fat percentage, milk fat yield, milk protein percentage, milk protein yield, and FE (kg milk produced/kg DMI). Because of the short and dynamic nature of the sampling periods, two time ranges which reflected steady consumption at 10% and 20% of NE_L_ were analyzed separately. Samples collected from d 3 and 4 of each period (while animals consumed top dressed feed at 10% of NE_L_ intake) were averaged and used to reflect “immediate” adaptation to the alternative top dress strategy. Samples collected from d 7 and 8 of each period (while animals consumed top dressed feed at 20% of NE_L_ intake) were also averaged and used to reflect “short-term” responses to the top dresses. All variables were analyzed in R version 3.5.2 [10] considering a replicated 4 × 4 Latin square design using a mixed-effects model with a top dress set as fixed effect and animal, period, and square designated as random effects. Tukey’s Least Square Differences were used for mean separation. Significant differences between treatments were declared at *p* < 0.05 and significant interactions were declared at *p* < 0.10.

## 3. Results and Discussion

### 3.1. Feed Composition

Composition of each feed ingredient was as expected with the notable exception of CG. The CP content of the CG was unrealistically high. To better understand if this CP content was truly representative of what animals were consuming, we back-calculated the CP content of the feed using the analyzed diet samples and the analyzed refusal samples. In each instance, we found similarities (<2% unit difference) in the estimated CP content of this top dress. Based on this exercise, it appears that the CG fed in this experiment was likely contaminated with a higher protein ingredient. As such, the results should not be taken to reflect responses associated with top dressed CG alone and should be assumed to represent top dressing with a CG, protein commodity mixture.

### 3.2. Dry Matter Intake Responses

Immediate and short-term DMI responses were affected by top dress type (*p* < 0.01; Table 5).

It is not surprising that top dress type affected DMI, as the top dresses differed in factors well-acknowledged to influence DMI [4]. These factors include NDF, starch, particle size, and palatability. Animals consuming diets top dressed with GH consumed less feed than cows on SBM, CG, or Control diets immediately and in the short-term (Table 5). The depression in intake associated with GH can be explained by the higher NDF and lower starch content of this top dress when compared to CG and SBM (Table 2). High NDF concentrations have previously been shown to downregulate feed intake by Mertens [11], who suggested that NDF can be used to predict DMI of dairy cows due to the positive correlation between NDF and the bulking density of feeds. Lower-starch diets have also been shown to decrease DMI in Holstein cows [12]. Additionally, the GH had larger particle size than the other treatments and likely had lower palatability. Larger particle size decrease DMI of lactating dairy cows [13], and ingestion of long fiber is well-linked with gut fill and reduced feed intake [14].

The DMI intake was also affected by the adaptation period (*p* < 0.01), where cows reduced DMI on average 1.63 kg in the short-term period compared to the immediate period (21.8 vs. 22.8, respectively), which could be explained by the gradual increase in the top dresses inclusion rate. Top dress interacted significantly with the adaptation period (*p* < 0.01), where DMI was not affected in Control, SBM, and CG top dresses, but it was reduced for GH, probably because its higher NDF content and particle size compared to the other treatments [4].

It is also likely that palatability played a role in the immediate responses of cows to the different top dress treatments. Immediate DMI of cows consuming CG was higher than the DMI of cows consuming the Control diets (24.7 vs. 23.1 kg/d DM basis; Table 5). Corn grain has been identified as a highly palatable feed for dairy cattle [15]; the increased DMI of the CG treatment in the immediate response time-frame is likely associated with the high palatability of CG. In the longer-term, feedback mechanisms have been identified that link higher starch diets with depressed intakes [16]. Starch is highly digestible and associated with rumen fermentation that increases propionate as a proportion of VFA absorbed [17]. Satiety signals that end feed intake in dairy cattle are likely caused by propionate, as propionate concentration in the liver is drastically increased during meals and then rapidly metabolized [16]. This rapid metabolism may have a suppressive effect on feed intake likely by stimulating oxidation of acetyl-CoA in the liver [18]. If present, this intake inhibition was likely already occurring by the short-term sampling range, especially since the concentration of CG in the diet would be at its highest during this period. In addition, feed intake in the higher-starch CG treatment was no longer different from the SBM or Control treatments (23.4 vs. 23.1 and 22.1 kg/d DM basis, respectively; Table 5). 

Feed intake of cows on the SBM diet did not differ from the Control diet in either the immediate or short-term response time-frames. This was somewhat unexpected because CP intake is commonly linked with increased DMI [19], and it was hypothesized that cows consuming SBM diets would have higher intakes than cows on Control diets due to the high CP concentration of the SBM top dress. However, it is possible that the composition of the basal TMR Only diet was already high enough in CP (Table 4) and that the additional provision associated with SBM intake did not result in altered intake regulation. The Control diet was formulated to contain 11.7% MP and 53 g RDP balance (DM basis). In a similar feeding situation, Imaizumi, et al. [20] reported that the addition of soybean and cottonseed meal did not affect DMI compared to a control diet with 15.9% CP and 10.8% MP (DM basis). Therefore, it is logical that the Control diet in the present study was likely high enough in CP that DMI was not affected by the addition of SBM.

It is worth noting that the random effect of animal was significant for DMI in both the immediate and the short-term response periods (*p* < 0.01 and *p* = 0.01, respectively; data not showed). Although animal effects can represent a number of factors including those directly associated with individual animal differences, they can also be easily confounded with other, less clear influences. In this case, the significance of animal effects is of interest because it highlights specific differences among animals that are repeatable across top dress types. Although not surprising because we expect animal genetic merit to play a major role in governing feed intake regulation [21], this significant difference among individuals across treatments is a challenge for individual animal feeding algorithms. If such algorithms are developed, the persistent differences among individuals suggest that model training or learning should occur at the individual animal level. This implies that data collected and presented in the literature on groups of animals may have minimal use in developing precision feeding algorithms that capitalize on individual variation. 

### 3.3. Milk Yield and Energy-Correct Milk

Milk yield was affected by top dress type (*p* < 0.01; Table 5) in the immediate and short-term response periods, and tended (*p =* 0.10) to be affected by the adaptation period (immediate vs. short term period). In both response periods, animals consuming CG and SBM treatments had greater MY than cows consuming GH or Control treatments (34.2 and 35.4 vs. 31.6 and 31.4 kg/d DM basis, respectively). The average increase in MY (0.72 kg/d) with the progression of the adaptation period might be explained by the increase in nutrient intake with the gradual increase of trop dresses like CG and SBM, since diets containing these two top dresses had less NDF and more starch and CP (Table 4). In the field of nutrition, convention in experimental design dictates that we focus on grouped animal responses and look at differences in responses after adaptation to diets; however, when attempting to derive methods for optimizing individual animal efficiency, there may be variation in the short-term that can be exploited. The increased MY of animals on the SBM diet, in particular, supports this idea because animals had elevated MY without having elevated DMI. Although this short-term increase in productivity may be minor in the context of annual milk production from a cow, future studies should focus on the within-animal repeatability of these types of temporal responses to feed alteration. If repeatable patterns are revealed, a cyclic feeding strategy or one with a number of short-term interventions could be designed to create “bumps” in productivity on a weekly or semi-monthly basis, which could maximize FE and animal performance. 

Immediate and short-term MY were also affected by initial performance (*p* < 0.01; data not showed); as would be expected, MY of individual cows during the experimental period were similar to yield values from the beginning of the study when DIM was controlled for in the model. These responses were expected because of previous knowledge about the effect of animal genetic merit [22] and the shape of the lactation curve [23]. The significance of initial responses suggests that individual cow genetics play an important role in governing MY. Given the moderate heritability of MY traits [22], it is not surprising that an animal’s MY prior to the trial was significantly associated with its MY during the trial.

Energy-corrected milk was affected by top dress type (*p* < 0.01; Table 5) in the immediate and short-term response periods, but not affected (*p* > 0.05) by the adaptation period (immediate vs. short term period). Similarly to milk yield, in both response periods, animals consuming CG and SBM treatments had greater ECM than cows consuming GH or Control treatments, with exception of CG and Control treatment, which did not differ in the short-term period. The similar ECM for CG and Control treatment might be explained by the greater milk fat percentage in the Control treatment in the short-term period compared to the CG treatment.

### 3.4. Milk Fat Percentage and Yield

During the immediate response period, milk fat percentage was not affected by top dress type (*p* = 0.24; Table 5) or animal (*p* *=* 0.19; data not showed) but was related to initial milk fat percentage (*p* < 0.01; data not showed). The lack of significant treatment effects on milk fat in the immediate term could be due to the time associated with regulating milk fat synthesis, or with the limited nutritional differences induced by the top dresses. Regulation of milk fat synthesis is associated with both digestive processes and tissue metabolism [24]. De novo fatty acid synthesis is inhibited by specific fatty acid intermediates that are produced in the mammary gland, and milk fat synthesis is limited by hydrolysis and biohydrogenation of lipids in the rumen [25]. The machinery regulating milk fat synthesis may not have responded to the dietary shifts of experimental treatments by 3 or 4 d. It has also been shown that diets depressing milk fat synthesis included large amounts of readily digestible carbohydrates and reduced amounts of fibrous materials [25]. This suggests that our experimental diets may have been within acceptable nutritive composition ranges that did not result in negative effects on milk fat synthesis in the immediate term due to the lower concentrations of energy coming from top dress during this period. The limited effect of animal and period on milk fat percentage was unexpected, as animal genetic merit [26] and stage of lactation [27] have been shown to influence milk fat percentages. It is possible that the individual animal significance was entirely explained by the significant initial performance effect and no additional individual animal variation in milk fat yield could be partitioned into the animal term of the model. Finally, it is also possible that the short-term nature of the study did not capture a long-enough snapshot of the lactation curve to demonstrate these expected differences.

In the short-term, milk fat percentage was affected by treatment (*p* < 0.01; Table 5) and initial performance (*p* < 0.01; data not showed). Diets top dressed with CG resulted in milk fat percentages similar to the GH and SBM top dressed diets, and were lower than milk fat percentages produced on the Control treatment (Table 5). In addition, top dress strategy interacted significantly with the adaptation period (*p* < 0.01), where diets top dressed with CG reduced milk fat percentage with the gradual increase of CG. The increased carbohydrate concentration and decreased fiber content of the experimental CG diet associated with the highest level of CG feeding (Table 4) was sufficient to induce minor changes in milk fat, which is consistent with our understanding of factors regulating milk fat synthesis [25]. The consistency of the effect of initial performance on short-term milk fat percentage suggests that individual animal differences in performance are likely to outweigh treatment-induced differences, providing further evidence for the need to develop databases on individual animal observations to enable precise and accurate feeding algorithms for individuals, rather than evaluating data obtained from groups of animals.

The difference between the immediate treatment effects on milk fat percentage and the short-term effects suggests that the time delay in regulating milk fat synthesis may be important to consider when designing strategies to capitalize on short-term responses of individual cows. Although MY responses may be more immediate, changing economically important factors like milk fat may require longer data collection periods for algorithm development and testing.

As expected, immediate and short-term milk fat yield results reflected the MY and milk fat percentage responses (Table 5). Top dress type affected milk fat yields in the immediate and short-term response periods (*p* < 0.01; Table 5). In the immediate time-scale, the Control treatment and the GH treatment had reduced milk fat yield compared to the groups supplemented with CG and SBM. This increase in milk fat yield is consistent with the elevated MY observed for CG and SBM treatment groups. During the short-term response period, the animals supplemented with GH had lower milk fat yield than the animals consuming any other treatment (Table 5). Again, this response was consistent with the depressed MY of cows consuming the GH treatment. The cows receiving the Control diets also had reduced MY, but this was counterbalanced by the elevated milk fat percentage of this group, resulting in no difference in milk fat yield compared to the CG and SBM treatment groups. Although not measured in this trial, we hypothesized that cows from Control diets might have had higher ruminal acetate production, the major substrate for milk fat synthesis [28], which might explain these outcomes. Despite of the interaction observed between trop dress type and adaptation period (*p =* 0.09), any meaningful comparison was significant, since within each treatment (top dress or control), any significant differences were detected when the immediate vs. short term period was compared (Table 5).

### 3.5. Milk Protein Percentage and Yield

Milk true protein percentage was not affected by top dress type within the immediate or short-term response periods (*p* > 0.77; Table 5). However, it was affected by the initial milk protein percentage in the immediate response period (*p* = 0.016; data not showed) and tended to be affected in the short-term response period (*p* = 0.08; data not showed). There was also a tendency of an increase in milk protein percentage from the immediate to the short-term period (*p* = 0.06), likely caused by the overall increase in protein intake when top dresses’ inclusion rates where increased [29]. An additional significant effect for the discrete, and random, animal term was observed (data not showed) in the immediate response period (*p* = 0.02), but not in the short-term response period (*p* = 0.38). The limited effect of treatment on milk protein percentage was not expected. The treatments were not designed to be isonitrogenous (Table 4), and therefore, it was expected that higher protein intake would drive elevated milk protein synthesis [29,30]. Certainly, the elevated MY responses on the SBM diet, in particular, highlighted that animals were sensitive to this treatment; however, this sensitivity did not confer changes in milk protein percentage. The lack of overall changes in milk protein percentage likely suggests that animals were already receiving adequate metabolizable protein supplies to facilitate peak milk protein synthesis. The relationship between initial milk protein percentage and the milk protein percentages observed throughout the trial clearly highlights the individual animal differences in milk protein synthesis and the consistency of those differences across dietary interventions. The change in the significance of the animal term between the immediate and the short-term response periods may reflect some latency of unique individuals to respond to dietary interventions. Another reason for this change in significance between the two timeframes may be due to the greater concentrations of energy in the diets provided by the various top dresses throughout the short-term response period.

Milk protein yield was affected by top dress type (*p* < 0.01; Table 5), initial milk protein yield and animal (*p* < 0.01; data not showed). Milk protein yield was also affected (*p =* 0.01) by the adaptation period (immediate vs. short-term period). The increase in milk protein yield was expected since both milk yield and milk protein percentage increased with the progression from immediate to short-term period. The supplemental SBM treatment resulted in greater milk protein yield compared with the other treatments during both periods. This elevated milk protein yield was most likely due to the elevated MY of cows consuming the supplemental SBM top dress. Feeding additional metabolizable protein through SBM top dress was likely a primary driver of elevated MY [31]. Indeed, diets supplemented with degradable protein have been shown to increase MY, but they do not consistently improve milk protein concentration [32]. Additionally, the effects of initial milk protein yield and animals further exemplify the importance of, and management potential associated with, obtaining data from and subsequently defining management strategies for individual animals. 

### 3.6. Feed Efficiency

Top dress type affected immediate and short-term FE (*p* < 0.01 and *p* = 0.03, respectively; Table 5). In the immediate response period, FE was highest for cows consuming top dressed GH and SBM; however, no statistical difference was identified between CG and Control diets or CG and SBM diets (Table 5). The increase in feed efficiencies of cows consuming supplemental SBM agrees with previous literature, as high protein supplements have been shown to increase FE [33]. In the short-term response period, FE was higher for cows consuming GH diets, and there were no differences in FE between CG, SBM, or Control diets (Table 5). Cows consuming GH top dress appeared to be more efficient due to the decrease in DMI. In the short-term, cows are able to support elevated MY despite low DMI, as they can leverage body reserves. However, if this strategy is employed in the long-term, downregulation of MY would likely be expected because cows would eventually adapt to low intake levels. In addition, cows would also be expected to reduce body condition with depressed DMI, which is generally undesirable. Feed efficiency was also affected (*p* = 0.01) by the adaptation period (immediate vs. short-term). The increase in FE in the short term was expected because of the average reduction in DMI associated with a tendency of increase in milk yield observed in this phase across treatments.

Immediate FE was also affected by initial performance and individual animal (*p* < 0.01; data not showed). Immediate feed efficiencies and their initial performance and animal effects are of great significance. These results suggest that there are short-term variations in individual cow feed efficiencies that can be used to maximize FE and, therefore, maximize profit for commercial dairy operations. However, the longer-term responses to this type of intervention and the repeatability of these responses across a full lactation curve is still unknown. The immediate responses shown in this study are the result of instantaneous responses to feeding high quality top dresses. 

### 3.7. Limitations and Considerations

Future research should focus on assessing these responses in a longer term i.e., an entire lactation. In addition, future studies should focus on the potential impacts of top-dressing lower quality feeds that likely come with lower costs, but also possible long-term health concerns such as toxicities and nutrient deficiencies. Finding an achievable balance between these strategies and assessing whether they are even worthwhile from an economic standpoint are warranted. 

More studies are also needed in order to increase the data available on immediate and short-term production responses to individual cow feeding strategies. In the big data era, a number of promising technologies are being developed that will probably change the way farms are operated and managed [3]. In this context, real-time forecasting of animal variables such as nutrient intake, MY, milk components and FE will be essential. The data collected in this study provide one small step toward developing models and algorithms to forecast productivity and FE and design custom supplements for individual cows. As individual cow data are more thoroughly leveraged from commercial farms and additional experimental studies on precision feed interventions are conducted, better datasets can be made available for model development. 

## 4. Conclusions

Immediate and short-term shifts in diet composition affected feed intake, MY and composition in this study (with the exception of milk protein percentage), and FE. The extent and duration of responses were top dress-dependent. Considerable variation across cows’ responses to the different top dresses fed was also identified. These results suggest an opportunity to use individualized feeding strategies to adjust dietary composition in the short-term to target economically optimal formulations without sacrificing production of individual dairy cows. However, substantially more data are needed before this goal will be achievable. 

## Figures and Tables

**Table 1:** Formulated composition of the common TMR fed to all treatment groups ^1^.

Ingredient	%, DM Basis
Corn silage, brown midrib	24.5
Alfalfa hay	2.16
Brewers grain	8.15
Corn grain, dry, ground	18.0
Cottonseed, whole, with lint	4.33
Milk cow concentrate	25.0
Soybean hulls, ground	5.49
Canola meal	4.03
Amino plus ^a^	3.93
Palmit 80 ^b^	0.86
Blood meal, dried	0.78
Sodium bicarbonate	0.50
Limestone, ground	0.50
Potassium carbonate	0.43
Salt, white	0.29
Volclay 90 ^c^	0.17
Molasses, cane	0.13
OmniGen-AF ^d^	0.17
Potassium magnesium sulfate	0.09
MHA, dry ^e^	0.08
Calcium phosphate, mono-dical	0.07
Mepron ^f^	0.06
Diamond XPC ^g^	0.04
Selenium yeast, 0.06%	0.04
Ultrasorb ^h^	0.03
Clarifly ^i^	0.03
Biotin ^j^	0.03
Zinpro 5 ^k^	0.02
Trace mineral blend	0.02
Vitamin A, D, E blend	0.01
Vitamin E premix, 60%	0.01
Rumensin 90 ^l^	0.003
TOTAL	100.0

^1^ TMR formulated to provide 61.7 mCal metabolizable energy (ME) per day; 2,750 g of metabolizable protein (MP) per day; 39.0 kg of ME allowable milk per day; and 39.0 kg of MP allowable milk per day. ^a^ Rumen protected soybean meal; Ag Processing, Inc; Omaha, NE, USA; ^b^ Vegetable palm oil; rumen bypass fat; ADM Animal Nutrition; Quincy, IL, USA; ^c^ Granular sodium bentonite; binder and digestive aid; American Colloid Company; Hoffman Estates, IL, USA; ^d^ Immune support supplement; microbial ingredients, vitamins, and aluminosilicates; Phibro Animal Health Corporation; Teaneck, NJ, USA; ^e^ Granular formulation of ALIMET; 84% methionine activity and 100% absorbed; NOVUS; Saint Charles, Missouri, USA; ^f^ Rumen protected DL-methionine; RP Nutrients, Inc; East Troy, WI, USA; ^g^ Saccharomyces cerevisiae fermentation product; Diamond V; Cedar Rapids, Iowa, USA; ^h^ Mycotoxin deactivator; Micron Bio-Systems Ltd.; Buena Vista, VA, USA; ^i^ Diflubenzuron feed-through fly control; Central Garden and Pet Company—Central Life Sciences; Schaumburg, IL, USA; ^j^ 2,200 mg Biotin/kg; ^k^ Trace mineral concentrate containing 30,688.51 ppm manganese, 5,370.57 ppm copper, 306.30 ppm iron, 75,187.97 ppm zinc, 613.32 ppm iodine, and 1,687.43 ppm cobalt (DM basis); Zinpro Performance Minerals; Eden Prairie, Minnesota; ^l^ Monensin sodium energy supplement for increased milk production efficiency; Elanco Animal Health; Greenfield, IN, USA.

**Table 2:** Chemical composition of TMR, CG, SBM, and GH feedstuffs (%, DM basis).

Item	TMR	Corn Grain (CG)	Soybean Meal (SBM)	Grass Hay (GH)
DM	46.7	84.3	81.6	92.8
CP	15.1	21.2 ^a^	51.7	6.80
NDF	39.7	13.4	9.80	73.8
ADF	22.1	3.70	4.60	44.3
Ash	6.10	4.30	10.1	7.20
Ether Extract	4.70	3.12	1.60	2.30
Starch	19.3	51.8	3.70	1.60
ADL	2.40	0.60	0.40	5.40

^a^ Suspected to be higher than typical values due to contamination during storage.

**Table 3:** Feedstuff inclusion rates in experimental treatment diets of TMR with and without the addition of CG, SBM, or GH (% of dietary net energy of lactation (NE_L_) intake).

Treatments ^1^	Feedstuffs			
	TMR	CG	SBM	GH
Days 3/4				
Control	100	0	0	0
CG	90	10	0	0
SBM	90	0	10	0
GH	90	0	0	10
Days 7/8				
TMR only	100	0	0	0
CG	80	20	0	0
SBM	80	0	20	0
GH	80	0	0	20

^1^ Treatments: Control = TMR with no top dress; CG = TMR with corn grain top dress; SBM = TMR with soybean meal top dress; GH = TMR with mixed grass hay top dress.

**Table 4:** Ingredient (kg/d, DM basis) and chemical composition of the experimental diets (% of dry matter unless otherwise stated).

Item	Treatments ^1^			
	Control	CG	SBM	GH
Composition				
Days 3/4				
TMR	26.4	24.0	23.2	23.4
CG		1.88		
SBM			1.87	
GH				2.99
Days 7/8				
TMR only	26.5	21.7	21.1	19.3
CG		3.82		
SBM			3.83	
GH				5.56
Chemical composition				
Days 3/4				
OM	93.9	94.0	93.6	93.9
ADF	22.1	20.8	20.8	25.1
NDF	39.7	37.8	37.5	44.1
Starch	19.3	21.7	22.0	17.5
Fat	4.73	4.62	4.50	4.67
CP	15.1	15.6	17.9	14.2
Days 7/8				
OM	93.9	94.1	93.2	94.0
ADF	22.1	19.3	19.2	27.7
NDF	39.9	35.3	31.8	48.5
Starch	19.3	24.2	25.2	15.9
Fat	4.73	4.49	4.21	4.61
CP	15.1	16.0	21.2	13.4

^1^ Treatments: Control = TMR with no top dress; CG = TMR with corn grain top dress; SBM = TMR with soybean meal top dress; GH = TMR with mixed grass hay top dress.

**Table 5:** Mean production performance responses of cows consuming experimental treatment diets of TMR with and without the addition of CG, SBM, or GH.

	Treatments ^2^						*p*-Values		
Response Variable (DM Basis)	Days ^1^	Control	CG	SBM	GH	SEM	Top Dress	ADP	Top Dress × ADP
DMI, kg/d	3/4	23.1 ^Ab^	24.7 ^Ac^	23.6 ^Abc^	20.1 ^Aa^	0.510	<0.01	<0.01	<0.01
	7/8	22.1 ^Ab^	23.4 ^Ab^	23.1 ^Ab^	16.4 ^Ba^	0.770	<0.01		
Milk yield, kg/d	3/4	31.4 ^a^	34.2 ^b^	35.4 ^b^	31.6 ^a^	0.950	<0.01	0.10	0.16
	7/8	33.0 ^a^	35.8 ^b^	36.3 ^b^	30.8 ^a^	0.990	<0.01		
Energy-corrected milk, kg/d	3/4	28.0 ^a^	30.4 ^b^	31.0 ^b^	28.0 ^a^	0.624	<0.01	0.16	0.18
	7/8	29.4 ^ab^	31.0 ^bc^	32.1 ^c^	27.1 ^a^	0.637	<0.01		
Milk fat, %	3/4	4.64 ^A^	4.63 ^A^	4.54 ^A^	4.61 ^A^	0.039	0.24	0.10	0.02
	7/8	4.68 ^Ab^	4.42 ^Ba^	4.61 ^Aab^	4.53 ^Aab^	0.064	<0.01		
Milk fat yield, kg/d	3/4	1.46 ^Aa^	1.58 ^Ab^	1.61 ^Ab^	1.46 ^Aa^	0.043	<0.01	0.42	0.09
	7/8	1.55 ^Ab^	1.56 ^Ab^	1.67 ^Ab^	1.40 ^Aa^	0.048	<0.01		
Milk true protein, %	3/4	2.92	2.91	2.91	2.89	0.047	0.96	0.06	0.83
	7/8	2.95	2.95	2.96	3.01	0.061	0.77		
Milk true protein yield, kg/d	3/4	0.922 ^a^	0.991 ^ab^	1.03 ^b^	0.915 ^a^	0.033	<0.01	0.01	0.63
	7/8	0.973 ^ab^	1.05 ^bc^	1.08 ^c^	0.930 ^a^	0.032	<0.01		
Feed efficiency, kg milk/kg feed	3/4	1.37 ^a^	1.39 ^ab^	1.49 ^bc^	1.58 ^c^	0.039	<0.01	0.01	0.13
	7/8	1.56 ^a^	1.56 ^a^	1.58 ^a^	2.28 ^b^	0.20	0.03		

^1^ Days 3/4 represent the immediate responses and days 7/8 represent the short-term responses. ^2^ Treatments: TMR Only = TMR with no top dress; CG = TMR with corn grain top dress; SBM = TMR with soybean meal top dress; GH = TMR with mixed grass hay top dress. ADP = Adaptation period (immediate and short-term responses); ^a–c^ Means within a row with different superscripts differ significantly from one another (*p* < 0.05); ^A,B^ Means within a column with different superscripts within each variable differ significantly from one another (Top Dress × ADP; *p* < 0.10).

## Data Availability

Data can be obtained from the corresponding author upon reasonable request to the corresponding author of the manuscript.

## Supplementary Materials

The following are available online at https://www.mdpi.com/article/10.3390/ani11123518/s1, Table S1: Individual cow milk yield, milk fat percentage, and milk protein percentage from monthly analyses.
