# Supplementary material for: Short-Term Adaptation of Dairy Cattle Production Parameters to Individualized Changes in Dietary Top Dress
Source: Animals (Basel). 2021 Dec 10;11(12):3518. doi: 10.3390/ani11123518 (PMC8697869; doi:10.3390/ani11123518)
Supplement: Supplementary file 1 [file animals-11-03518-s001.zip › animals-1471184-Supplementary.pdf]

Supplementary Material

Table S1. Individual cow milk yield, milk fat percentage, and milk protein percentage from monthly analyses.

Cow ID	Lactation #	Calving Date	DIM	Analysis Date	MY, kg/d	Milk Fat, %	Milk Protein, %
5096	4	9/8/2018	164	2/18/2019	40.6	6.6	3.4
5096	4	9/8/2018	192	3/18/2019	32.9	5.2	3.6
5175	4	7/15/2018	219	2/18/2019	34.7	4.3	3.6
5175	4	7/15/2018	247	3/18/2019	36.9	4	3.5
5225	4	8/21/2018	182	2/18/2019	36.6	5.3	3.7
5225	4	8/21/2018	210	3/18/2019	41.2	4.9	3.6
5293	3	8/4/2018	199	2/18/2019	33.6	4.6	3.6
5293	3	8/4/2018	227	3/18/2019	27.5	4.3	3.6
5294	3	6/27/2018	237	2/18/2019	30.2	5.5	3.3
5294	3	6/27/2018	265	3/18/2019	30.5	4.3	3.4
5320	3	8/22/2018	181	2/18/2019	40.6	3.8	3.6
5320	3	8/22/2018	209	3/18/2019	34.7	4.1	3.3
5333	3	7/5/2018	229	2/18/2019	29.5	4.6	3.8
5333	3	7/5/2018	257	3/18/2019	25.3	4.2	3.4
5421	2	8/25/2018	178	2/18/2019	41.2	4.9	3.2
5421	2	8/25/2018	206	3/18/2019	36.4	4.1	3.2
5459	2	7/7/2018	255	3/18/2019	18.6	5.4	3.8
5463	2	6/27/2018	237	2/18/2019	26.8	6.1	3.6
5463	2	6/27/2018	265	3/18/2019	29.5	5.9	3.8
5476	2	7/30/2018	204	2/18/2019	44.4	3.8	3.4
5476	2	7/30/2018	232	3/18/2019	38.1	3.6	3.4
5479	2	8/14/2018	189	2/18/2019	44.2	5.1	3
5479	2	8/14/2018	217	3/18/2019	44.1	4.3	3.1
5555	1	7/30/2018	204	2/18/2019	34.6	4	3.3
5555	1	7/30/2018	232	3/18/2019	35.2	3.9	3.1
5558	1	7/4/2018	230	2/18/2019	30.2	5.2	3.9
5558	1	7/4/2018	258	3/18/2019	28.1	5.2	3.8
5563	1	7/30/2018	204	2/18/2019	38.6	5.6	3.1
5563	1	7/30/2018	232	3/18/2019	35.5	4	3.2
5568	1	7/4/2018	230	2/18/2019	35.3	5.4	3.4
5568	1	7/4/2018	258	3/18/2019	33.1	5.1	3.5
5578	1	7/25/2018	209	2/18/2019	22.8	5.8	3.8
5578	1	7/25/2018	237	3/18/2019	21.0	5.8	3.8
5580	1	8/13/2018	190	2/18/2019	32.7	4.3	3.4
5580	1	8/13/2018	218	3/18/2019	30.9	4.4	3.2
5585	1	8/23/2018	180	2/18/2019	34.5	3.7	3.1
5585	1	8/23/2018	208	3/18/2019	31.2	3.8	2.5

5587	1	9/16/2018	156	2/18/2019	25.9	4.9	3.7
5587	1	9/16/2018	184	3/18/2019	28.6	5	3.7
5601	1	6/29/2018	235	2/18/2019	42.7	4	3.4
5601	1	6/29/2018	263	3/18/2019	43.6	4.2	3.3
5603	1	7/25/2018	209	2/18/2019	37.5	4.3	2.8
5603	1	7/25/2018	237	3/18/2019	34.5	3.9	3
5611	1	7/27/2018	207	2/18/2019	30.0	4.8	3.5
5611	1	7/27/2018	235	3/18/2019	28.6	4.1	3.5
5617	1	7/24/2018	210	2/18/2019	32.3	4.9	3.5
5617	1	7/24/2018	238	3/18/2019	29.4	5.4	3.2
